# Longitudinal risk of maternal hospitalization for mental illness following preterm birth

**DOI:** 10.1186/s12916-022-02659-9

**Published:** 2022-11-17

**Authors:** Gabriel Côté-Corriveau, Gilles Paradis, Thuy Mai Luu, Aimina Ayoub, Marianne Bilodeau-Bertrand, Nathalie Auger

**Affiliations:** 1 Department of Epidemiology, Biostatistics, and Occupational Health, McGill University, Montreal, Quebec Canada; 2 Institut national de santé publique du Québec, 190 Cremazie Blvd E., Montreal, Quebec H2P 1E2 Canada; 3 Department of Pediatrics, Sainte-Justine Hospital Research Centre, University of Montreal, Montreal, Quebec Canada; 4 University of Montreal Hospital Research Centre, Montreal, Quebec Canada; 5 School of Public Health, University of Montreal, Montreal, Quebec Canada

**Keywords:** Longitudinal studies, Maternal mental illness, Postpartum period, Pregnancy, Preterm birth

## Abstract

**Background:**

Preterm birth may affect maternal mental health, yet most studies focus on postpartum mental disorders only. We explored the relationship between preterm delivery and the long-term risk of maternal hospitalization for mental illness after pregnancy.

**Methods:**

We performed a longitudinal cohort study of 1,381,300 women who delivered between 1989 and 2021 in Quebec, Canada, and had no prior history of mental illness. The exposure was preterm birth, including extreme (<28 weeks), very (28-31 weeks), and moderate to late (32-36 weeks). The outcome was subsequent maternal hospitalization for depression, bipolar, psychotic, stress and anxiety, personality disorders, and self-harm up to 32 years later. We used adjusted Cox proportional hazards models to estimate hazard ratios (HR) and 95% confidence intervals (CI) for the association between preterm birth and mental illness hospitalization.

**Results:**

Compared with term, women who delivered preterm had a higher rate of mental illness hospitalization (3.81 vs. 3.01 per 1000 person-years). Preterm birth was associated with any mental illness (HR 1.38, 95% CI 1.35-1.41), including depression (HR 1.37, 95% CI 1.32-1.41), psychotic disorders (HR 1.35, 95% CI 1.25-1.44), and stress and anxiety disorders (HR 1.42, 95% CI 1.38-1.46). Delivery at any preterm gestational age was associated with the risk of mental hospitalization, but risks were greatest around 34 weeks of gestation. Preterm birth was strongly associated with mental illness hospitalization within 2 years of pregnancy, although associations persisted throughout follow-up.

**Conclusions:**

Women who deliver preterm may be at risk of mental disorders in the short and long term.

**Supplementary Information:**

The online version contains supplementary material available at 10.1186/s12916-022-02659-9.

## Background

Preterm birth is a frequent pregnancy complication [1], but the impact on maternal mental health is unclear. Preterm delivery is associated with neonatal morbidity, neurodevelopmental impairment, and adverse childhood outcomes that may be difficult for parents to manage [1, 2]. Several studies have shown that extreme prematurity can affect maternal mental health, including depression, anxiety, and posttraumatic stress disorders [3–5]. The majority of studies investigate extreme and very preterm birth, focusing on women who deliver before 32 weeks of gestation. Yet women who deliver very early are the minority, as most deliver between 32 and 36 weeks at moderate to late gestational ages [1]. Although moderate to late preterm birth leads to neonatal complications less frequently, children may nevertheless develop longstanding morbidities that could affect maternal mental health [2].

Studies are beginning to suggest that moderate preterm birth may impact maternal mental health [6–8]. A population-based study of 89,366 women found that moderate to late preterm birth was linked with feelings of hopelessness in the postpartum period [7]. Symptoms of postpartum depression and posttraumatic stress were also prevalent in cohorts of women who delivered moderate to late preterm [6, 8]. However, the majority of these studies were limited by small samples of women, lack of attention to outcomes beyond stress and depression, and little to no follow-up past the postpartum period [3, 4, 6–8]. As the influence of preterm birth may continue throughout childhood and impact parents over the long term [5, 9, 10], we assessed the relationship between the severity of preterm birth and risk of maternal hospitalization for a range of mental disorders over 32 years of follow-up.

## Methods

### Study population

We conducted a longitudinal cohort study of 1,381,300 women who delivered in hospitals in Quebec, Canada, between 1989 and 2021. We identified women at their first delivery and included all subsequent deliveries. We compiled the cohort using the Maintenance and Use of Data for the Study of Hospital Clientele registry. The registry is updated annually and has all discharge summaries for delivery and mental illness hospitalizations, including up to 41 diagnostic and 35 procedure codes. The robustness of the registry has been established [11]. As more than 98% of women deliver in hospital, the registry covers nearly all deliveries in the province. For women who delivered in 1989, there are up to 32 years of follow-up.

### Inclusion/exclusion criteria

We included all women who delivered at 22 weeks or more of gestation, the limit of viability [12]. We excluded women with invalid health insurance numbers because they could not be tracked over time, and women with a history of mental illness during pregnancy or prior hospitalizations.

### Preterm birth

The main exposure measure was preterm birth, defined as less than 37 weeks of gestation. Gestational age was determined mainly by dating ultrasound in the first trimester. We categorized preterm birth as binary (preterm, term) in primary analyses. We further considered the degree of prematurity, including extreme (<28 weeks), very (28-31 weeks), and moderate to late (32-36 weeks). We also expressed gestational age as a continuous exposure using restricted cubic splines [13].

### Maternal mental illness

The outcome was maternal mental illness requiring hospitalization after delivery. Follow-up for mental health hospitalizations began at the first delivery for all women, and ended at the first mental health hospitalization, death, or the end of study on March 31, 2021. Outcomes included depression, bipolar disorder, psychotic disorder, stress and anxiety disorder, personality disorder, and intentional self-harm. Intentional self-harm mainly included suicide attempts, as hospitalization for nonsuicidal self-harm is infrequent in Quebec. We used codes from the *International Statistical Classification of Diseases and Related Health Problems, Ninth and Tenth Revisions* (*ICD-9* and *ICD-10*) to identify mental illness hospitalizations anywhere in Quebec (Additional file 1: Table S1). Mental disorder classification in the ICD follows the *Diagnostic and Statistical Manual of Mental Disorders, Fifth Edition (DSM-5)*.

### Covariates

We accounted for factors associated with preterm birth and mental illness that could represent confounders [10, 14–17]. Covariates included age at first delivery (<25, 25–34, ≥35 years); comorbidity defined as preexisting obesity, diabetes, hypertension, or dyslipidemia; substance use disorder defined as alcohol, tobacco, or illicit drug use; gestational diabetes; severe maternal morbidity including severe preeclampsia, cerebrovascular accidents, cardiac complications, and other life-threatening complications [18]; cesarean section; multiple birth; fetal congenital anomaly; socioeconomic deprivation defined as the most disadvantaged quintile of the population according to neighborhood income, employment, and education [19]; rurality (yes, no, unspecified); and time period (1989-1998, 1999-2009, 2010-2021). We measured covariates at the first delivery using the *ICD*, *Canadian Classification of Diagnostic, Therapeutic, and Surgical Procedures*, and *Canadian Classification of Health Interventions* [14, 18].

### Data analysis

We computed hospitalization rates for maternal mental illness per 1000 person-years and plotted the cumulative incidence over time [20]. In primary analyses, we used Cox proportional hazards regression models to estimate hazard ratios (HR) and 95% confidence intervals (CI) for the association between preterm birth and maternal hospitalization for mental illness. HRs represent the risk of hospitalization for women who deliver preterm during a specified time interval relative to women who deliver at term. We defined the time scale as the number of days since first delivery. We handled deaths as a competing event and censored women who were not hospitalized for mental illness at the end of follow-up.

As women could have more than one pregnancy, we accounted for subsequent deliveries by analyzing preterm birth as a time-varying exposure. We classified women as exposed from their first preterm delivery, with follow-up beginning at the first pregnancy. Women with a preterm delivery remained exposed until the end of the study regardless of subsequent deliveries. Women who never had a preterm delivery in any pregnancy were considered unexposed for the entire study period. We adjusted the models for age at first delivery, comorbidity, substance use disorder, gestational diabetes, severe maternal morbidity, cesarean section, multiple birth, fetal congenital anomaly, socioeconomic deprivation, rurality, and time period.

In secondary analyses, we assessed whether the associations differed according to the degree of prematurity at the first delivery. To do so, we ran regression models with gestational age on a continuous scale in splines [13]. We placed knots at 28, 32, 36, 40, and 42 weeks of gestation and used 40 weeks as the reference. We assessed the proportionality of hazards using cumulative incidence curves and used flexible parametric models to determine if HRs varied over the duration of follow-up [21].

We performed sensitivity analysis with preterm birth as a fixed exposure at the first delivery. We also examined how categories of preterm birth, including extreme, very, and moderate to late, were associated with maternal mental illness hospitalization.

We performed the analysis in SAS version 9.4 (SAS Institute Inc., Cary, NC) and assessed statistical significance with 95% CIs. The institutional review board of the University of Montreal Hospital Centre provided an ethics waiver as the data were anonymous and informed consent was deemed not necessary.

## Results

### Incidence of maternal mental illness

Among 1,381,300 women who delivered between 1989 and 2021, 99,411 (7.2%) had a preterm birth (Additional file 1: Table S2). During 23,841,686 person-years of follow-up, 6362 women with preterm birth (6.4%) and 66,789 women with term birth (5.2%) were later hospitalized for mental illness (Table 1). Average time between the first delivery and hospitalization for mental illness was 12.4 years (SD 8.1). The rate of mental illness hospitalization was higher for preterm than term delivery (3.81 vs. 3.01 per 1000 person-years) but was greatest for delivery between 28 and 31 weeks (3.98 per 1000 person-years). After 32 years of follow-up, the cumulative incidence of maternal mental illness hospitalization was 119.9 per 1000 preterm births, compared with 96.8 per 1000 term births.

**Table 1:** Incidence of maternal mental illness hospitalization according to severity of preterm birth

	Total no. of women	No. of women hospitalized for mental illness	Incidence per 1000 person-years (95% confidence interval)	Cumulative incidence per 1000 women at 32 years of follow-up (95% confidence interval)
Preterm birth, weeks				
<37	99,411	6362	3.81 (3.72-3.90)	119.9 (113.1-126.9)
<28	6716	399	3.84 (3.48-4.23)	116.0 (102.6-130.3)
28-31	8866	581	3.98 (3.67-4.32)	119.8 (107.0-133.3)
32-36	83,829	5382	3.79 (3.69-3.89)	120.1 (112.4-128.1)
≥37	1,281,889	66,789	3.01 (2.99-3.04)	96.8 (95.8-97.9)

### Cumulative incidence of mental illness

**Fig. 1:**
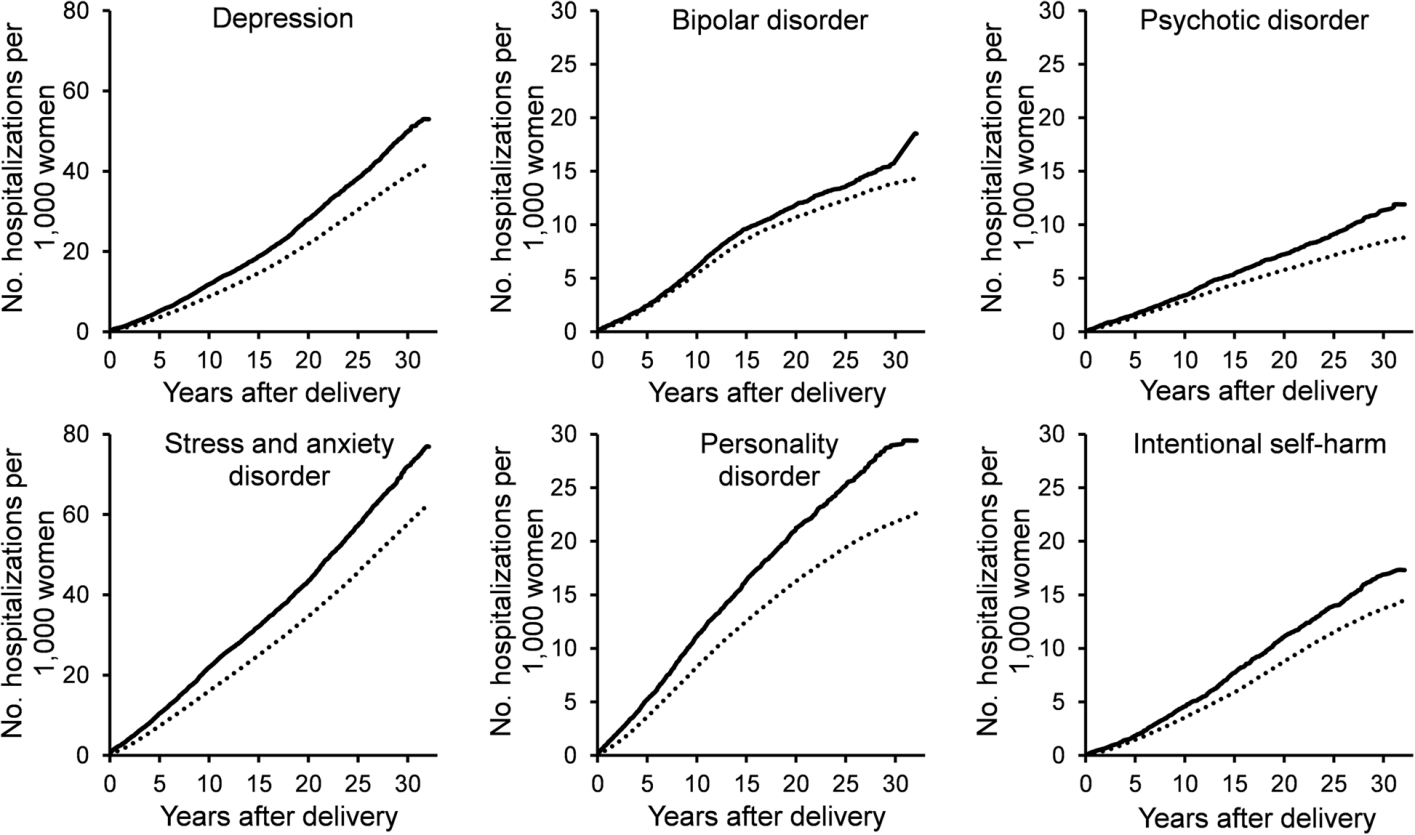
Cumulative incidence of mental illness hospitalization among women with preterm birth (black line) and without preterm birth (dotted line)

Cumulative incidence rates were higher among women with preterm delivery regardless of the type of mental disorder (Fig. 1). At 32 years of follow-up, rates were greatest for stress and anxiety disorders, with 76.9 admissions per 1000 preterm deliveries versus 62.3 per 1000 term deliveries. Hospitalization rates for stress and anxiety and personality disorders started increasing immediately after delivery for women with preterm birth. For other mental disorders, the increase became more apparent around 10 years after pregnancy.

### Association between preterm birth and maternal mental illness

In adjusted regression models, women who delivered preterm had a higher risk of any mental disorder hospitalization (Table 2). Overall, preterm delivery was associated with 1.38 times the risk of any mental illness hospitalization (95% CI 1.35-1.41) relative to term birth. Preterm birth was associated with around 1.4 times the risk of depression (HR 1.37, 95% CI 1.32-1.41), psychotic disorder (HR 1.35, 95% CI 1.25-1.44), stress and anxiety disorder (HR 1.42, 95% CI 1.38-1.46), personality disorder (HR 1.50, 95% CI 1.44-1.56), and intentional self-harm (HR 1.36, 95% CI 1.28-1.43). The association with bipolar disorder was slightly less pronounced.

**Table 2:** Association between preterm birth and maternal mental illness hospitalization

	No. of women		Incidence per 1000 person-years (95% confidence interval)		Hazard ratio (95% confidence interval)	
	Preterm	Term	Preterm	Term	Unadjusted	Adjusted^a^
Any mental illness	6362	66,789	3.81 (3.72-3.90)	3.01 (2.99-3.04)	1.45 (1.42-1.48)	1.38 (1.35-1.41)
Depression	2573	26,651	1.50 (1.45-1.56)	1.18 (1.16-1.19)	1.45 (1.40-1.50)	1.37 (1.32-1.41)
Bipolar disorder	974	11,409	0.56 (0.53-0.60)	0.50 (0.49-0.51)	1.24 (1.17-1.30)	1.22 (1.15-1.29)
Psychotic disorder	639	6524	0.37 (0.34-0.40)	0.29 (0.28-0.29)	1.39 (1.29-1.48)	1.35 (1.25-1.44)
Stress and anxiety disorder	3977	41,456	2.34 (2.27-2.42)	1.85 (1.83-1.86)	1.49 (1.46-1.53)	1.42 (1.38-1.46)
Personality disorder	1772	17,623	1.03 (0.99-1.08)	0.78 (0.77-0.79)	1.59 (1.53-1.66)	1.50 (1.44-1.56)
Intentional self-harm	932	9930	0.54 (0.51-0.58)	0.44 (0.43-0.44)	1.47 (1.39-1.55)	1.36 (1.28-1.43)

^a^Hazard ratio for preterm vs. term, adjusted for age, comorbidity, substance use disorder, gestational diabetes, severe maternal morbidity, cesarean section, multiple birth, fetal congenital anomaly, socioeconomic deprivation, rurality, and time period

### Effect of continuous gestational age

**Fig. 2:**
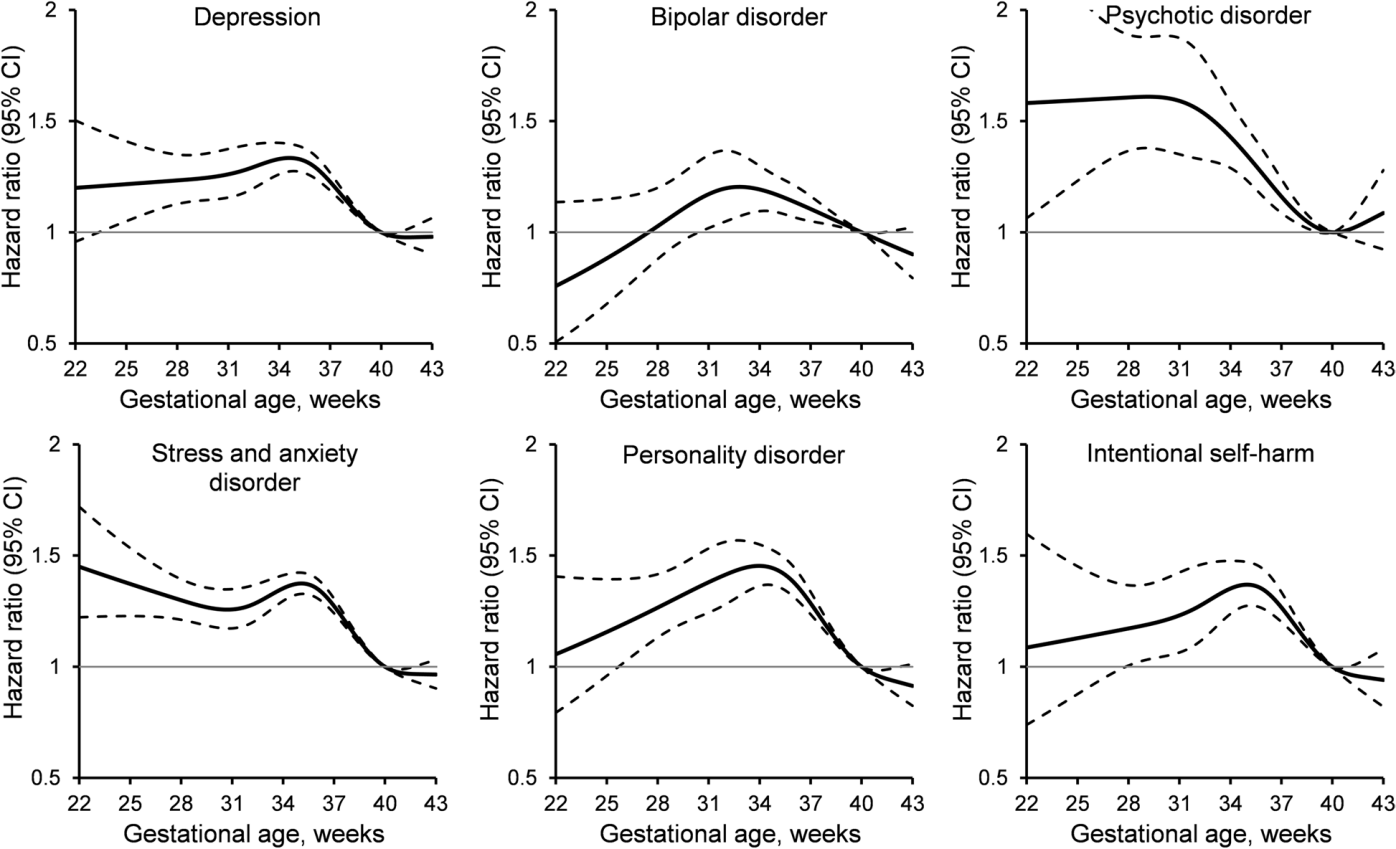
Association of gestational age with risk of mental illness hospitalization any time during follow-up. Hazard ratio (solid line) and 95% confidence interval (dashed line) for gestational age on a continuous scale relative to 40 weeks, adjusted for age, comorbidity, substance use disorder, gestational diabetes, severe maternal morbidity, cesarean section, multiple birth, fetal congenital anomaly, socioeconomic deprivation, rurality, and time period

In analyses of gestational age on a continuous scale, risk of mental illness hospitalization was greater for very and moderate to late preterm birth (Fig. 2). Compared with 40 weeks, delivery at 34 weeks of gestation was associated with 1.33 times the risk of depression (95% CI 1.26-1.40), 1.19 times the risk of bipolar disorder (95% CI 1.09-1.30), 1.35 times the risk of stress and anxiety disorder (95% CI 1.30-1.41), 1.45 times the risk of personality disorder (95% CI 1.36-1.55), and 1.35 times the risk of intentional self-harm (95% CI 1.24-1.48). Risk of psychotic disorder was greater at 30 weeks of gestation, with 1.61 times the risk of mental illness hospitalization (95% CI 1.37-1.88) compared with delivery at 40 weeks.

### Timing of mental illness after delivery

**Fig. 3:**
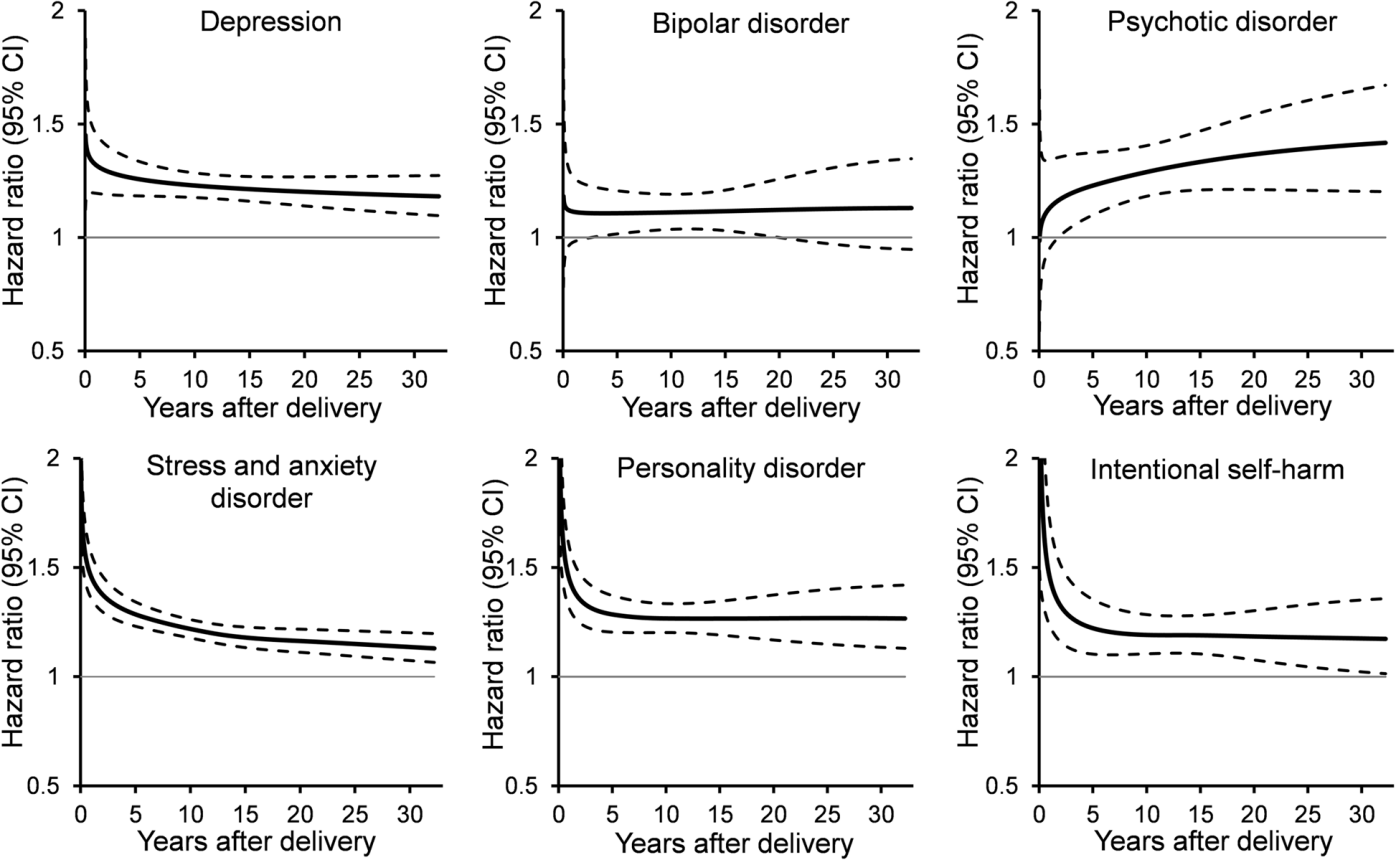
Association of preterm birth with mental illness hospitalization according to number of years after delivery. Hazard ratio (solid line) and 95% confidence interval (dashed line) for preterm vs. term, adjusted for age, comorbidity, substance use disorder, gestational diabetes, severe maternal morbidity, cesarean section, multiple birth, fetal congenital anomaly, socioeconomic deprivation, rurality, and time period

Preterm birth was associated with mental illness hospitalization throughout follow-up, although HRs were strongest within 1 to 2 years of delivery (Fig. 3). At 1 year postpartum, preterm birth was associated with 1.32 times the risk of depression (95% CI 1.19-1.46) and 1.45 times the risk of stress and anxiety disorder (95% CI 1.36-1.56), personality disorder (95% CI 1.31-1.59), and intentional self-harm (95% CI 1.24-1.72). Five years after delivery, preterm birth was associated with 1.26 times the risk of depression (95% CI 1.18-1.33), 1.29 times the risk of stress and anxiety disorder (95% CI 1.23-1.34), 1.29 times the risk of personality disorder (95% CI 1.20-1.37), and 1.22 times the risk of intentional self-harm (95% CI 1.10-1.35). The association with psychotic disorder increased gradually over time, with 1.42 times the risk of hospitalization at 32 years of follow-up (95% CI 1.20-1.67).

### Sensitivity analyses

In sensitivity analyses, models with preterm birth as a time-fixed exposure yielded slightly weaker associations (Additional file 1: Table S3). In analyses of preterm birth stratified by severity, extreme (HR 1.24, 95% CI 1.13-1.37), very (HR 1.22, 95% CI 1.13-1.33), and moderate to late preterm birth (HR 1.23, 95% CI 1.19-1.26) were all associated with the risk of mental health hospitalization relative to term (Additional file 1: Table S4).

## Discussion

In this cohort of 1.4 million women, preterm delivery was associated with a higher risk of hospitalization for mental illness up to 32 years after pregnancy. Risk of hospitalization was greatest within 2 years of delivery, but persisted throughout follow-up. Associations were present with all types of mental disorders, including depression, stress and anxiety disorders, and personality disorders. Women with moderate to late preterm birth were as much at risk of mental disorder hospitalization as women with extreme and very preterm birth. For some mental disorders, risks were even greater around 34 weeks of gestation or moderate preterm delivery. The findings suggest that women who deliver preterm may be at risk of hospitalization for a range of mental disorders in the short and long term, even when delivery occurs at moderate to late gestational ages.

Few studies have addressed moderate preterm birth in maternal mental health. Most of the literature focuses on depression or stress disorders in women with extreme or very preterm delivery [3, 22, 23]. Very preterm delivery is associated with 1.4 to 2.9 times the risk of postpartum depression compared with term delivery [3, 22]. Up to 22% of women who deliver before 30 weeks develop posttraumatic stress the first year postpartum [23]. Nevertheless, effects may not be limited to extreme or very preterm birth. A study of 91 mothers who delivered moderate to late preterm reported that symptoms of depression, anxiety, and post-traumatic stress were elevated up to 6 months later [6]. In population-based studies from Sweden and the United States, women who delivered between 32 and 36 weeks of gestation had 1.2 times the risk of postpartum depression compared with term [7, 22]. A study of 60 participants suggested that women who deliver moderate to late preterm have more postpartum depressive symptoms than women who deliver very preterm [8].

In our study, risk of maternal mental disorders was greatest for deliveries around 34 weeks of gestation, which is consistent with the few available studies [6–8, 22]. However, the association with stress, anxiety, and psychotic disorders was also present at lower gestational ages, suggesting that preterm birth may affect anxiety-linked mental health disorders differently. Extremely premature infants have a high prevalence of morbidity and developmental delay [1], which may be stressful for caregivers. While moderate to late preterm birth is associated with less morbidity, most support programs do not cover these births [24]. These factors may explain why any degree of preterm birth is associated with stress, anxiety, and psychotic disorders. In contrast, inadequate social support for mothers with moderate to late preterm birth may be more likely to lead to other types of mental disorders.

Longitudinal studies are conflicting for outcomes after the postpartum period. In a cohort of 214 mothers, very preterm delivery was associated with anxiety and depressive symptoms 7 years after birth [5]. Another study found that women who delivered very preterm were at risk of anxiety and depression 13 years later [10]. Three separate analyses of a total of 726 women found no association between very preterm delivery and depression, stress, or anxiety up to 25 years later [9, 25, 26]. The authors suggested that the impact of preterm delivery faded by the time children reach adolescence [9, 26]. However, the number of women studied was low. Our analysis of 1.4 million mothers suggests that women who deliver preterm remain at risk of mental illness up to 32 years later. The effects weakened over time but did not disappear.

Research has focused more on maternal depression and stress than other mental disorders [3–10, 22, 23, 25, 26]. Two Swedish cohort studies reported that preterm delivery increased the risk of psychosis 3 months postpartum [27, 28]. In one report, preterm delivery was not associated with suicide attempts the first year postpartum [29]. In contrast, we found that preterm birth was associated with up to 1.5 times the risk of most mental disorders later in life. The low number of cases, narrow range of mental disorders, and short follow-up may explain why previous studies differed from ours.

Preterm birth was associated with stress, anxiety, and personality disorders shortly after delivery, while the impact on depression, psychotic, and bipolar disorders appeared only 10 years later. Immediate consequences of preterm birth, including infant morbidity and need for neonatal intensive care, may be associated with maternal stress and anxiety in the short term. Childhood health and behavioral disorders that occur later may impact maternal depression, psychotic, and bipolar disorders in the long term. Data indicate that parental distress due to caring for a preterm infant may change as a child grows [5]. Respiratory and gastrointestinal disorders may be more concerning during infancy, while neurodevelopmental disability and behavioural problems may become prevalent during childhood and adolescence [2].

Maternal complications leading to preterm delivery may also be associated with mental disorders. Obesity, gestational diabetes, and preeclampsia increase the risk of preterm delivery [15] and are associated with postpartum mental illness [14, 17, 30]. Severe maternal morbidity, including severe preeclampsia, cerebrovascular accidents, and other life-threatening complications, is associated with 1.7 times the risk of mental illness the first year postpartum [16]. Pregnancy complications share immuno-inflammatory pathophysiology with mental illness, including dysregulation of the hypothalamic-pituitary-adrenal axis [17, 30, 31]. In our study, preterm birth was associated with maternal mental health despite adjustment for pregnancy complications. Thus, it is unlikely that maternal morbidity fully explains the association between preterm delivery and mental disorders.

This study has limitations. The outcome included severe maternal mental illness that required hospitalization, not mild problems that never led to hospitalization. We excluded women with mild or severe mental illness at the start of follow-up but could not identify mild disorders that were not reported. As we used administrative data, nondifferential misclassification may have occurred due to coding errors. We were not able to investigate associations for planned versus spontaneous preterm delivery. We could not account for childhood morbidity or psychiatric comorbidity. Although we controlled for several confounders, we cannot rule out residual confounding as we had no data on ethnicity, marital status, social support, psychotropic medications, or psychotherapy. A confounder would have to be associated with at least two times the risk of preterm birth and mental illness to fully confound our associations [32]. Quebec is a multicultural province, but studies across diverse countries, cultures, and ethnicities are needed before generalizing at a national level.

## Conclusions

In this longitudinal study of 1.4 million women followed for up to 32 years, preterm delivery was associated with an increased risk of hospitalization for several mental conditions, including stress and anxiety disorder, depression, and psychotic disorder. Women who delivered preterm had an elevated risk of mental health hospitalization whether they delivered extreme, very, or moderate to late preterm. Risk of most mental disorders was greatest within 2 years of preterm delivery, but remained high thereafter. The study provides novel evidence that women who deliver preterm are at risk of mental health disorders beyond the postpartum period. Public health policies should include long-term mental health support for all women who deliver preterm. Clinicians caring for preterm children should be aware of the potential mental health needs of mothers.

## Supplementary Information

**Additional file 1: Table S1.** Diagnostic codes for mental disorders. **Table S2.** Characteristics of women with and without preterm birth at first delivery. **Table S3**. Association between preterm birth at first delivery and maternal mental illness hospitalization. **Table S4.** Association between severity of preterm birth at first delivery and maternal mental illness hospitalization.

## Data Availability

The dataset supporting the findings of this study is available from the Ministry of Health and Social Services upon reasonable request.

## Abbreviations

CI: Confidence interval
HR: Hazard ratio
ICD: International Statistical Classification of Diseases and Related Health Problems
SD: Standard deviation
