# Supplementary material for: Longitudinal risk of maternal hospitalization for mental illness following preterm birth
Source: BMC Med. 2022 Nov 17;20:447. doi: 10.1186/s12916-022-02659-9 (PMC9670637; doi:10.1186/s12916-022-02659-9)
Supplement: Supplementary file 1 — Additional file 1: Table S1. Diagnostic codes for mental disorders. Table S2. Characteristics of women with and without preterm birth at first delivery. Table S3. Association between preterm birth at first delivery and maternal mental illness hospitalization. Table S4. Association between severity of preterm birth at first delivery and maternal mental illness hospitalization. [file 12916_2022_2659_MOESM1_ESM.docx]

**Table S1** Diagnostic codes for mental disorders

|  | International Classification of Diseases 9th Revision | International Classification of Diseases 10th Revision |
| --- | --- | --- |
| Depression | 296.2, 296.3, 298.0, 300.4, 311 | F32, F33, F34.1, F53.0 |
| Bipolar disorder | 296.0, 296.1, 296.4-296.8, 298.1, 301.13 | F30, F31, F34.0 |
| Psychotic disorder | 295, 297, 298.2-298.4, 298.8, 298.9 | F20-F29, F53.1 |
| Stress and anxiety disorder | 300.0-300.3, 300.5-300.9, 308, 309 | F40-F48 |
| Personality disorder | 301.0, 301.10-301.12, 301.2-301.9 | F60, F61 |
| Intentional self-harm | E950-E959 | X60-X84, Y87.0, Z91.5 |

**Table S2** Characteristics of women with and without preterm birth at first delivery

|  | No. women (%) | |
| --- | --- | --- |
|  | Preterm birth (N=99,411) | Term birth (N=1,281,889) |
| Age at first delivery, years |  |  |
| <25 | 25,801 (25.9) | 327,866 (25.6) |
| 25-34 | 60,019 (60.4) | 809,694 (63.2) |
| ≥35 | 13,591 (13.7) | 144,329 (11.2) |
| Comorbidity^a^ | 5,793 (5.8) | 47,950 (3.7) |
| Substance use disorder | 2,054 (2.1) | 18,943 (1.5) |
| Gestational diabetes | 6,722 (6.8) | 65,864 (5.1) |
| Cesarean section | 31,819 (32.0) | 276,466 (21.6) |
| Severe maternal morbidity^b^ | 10,754 (10.8) | 28,715 (2.2) |
| Multiple birth | 10,363 (10.4) | 9,100 (0.7) |
| Fetal congenital anomaly | 5,842 (5.9) | 33,548 (2.6) |
| Socioeconomic deprivation | 19,921 (20.0) | 240,945 (18.8) |
| Rural place of residence | 17,033 (17.1) | 212,400 (16.6) |
| Time period at delivery |  |  |
| 1989-1998 | 38,696 (38.9) | 526,490 (41.1) |
| 1999-2009 | 30,929 (31.1) | 371,807 (29.0) |
| 2010-2021 | 29,786 (30.0) | 383,592 (29.9) |
| Total person-years | 1,669,862 | 22,171,824 |

^a^Preexisting obesity, diabetes, hypertension, or dyslipidemia.

^b^Severe preeclampsia, cerebrovascular accident, and other life-threatening complications.

**Table S3** Association between preterm birth at first delivery and maternal mental illness hospitalization

|  | Hazard ratio  (95% confidence interval) | | |
| --- | --- | --- | --- |
|  | Unadjusted | Adjusted^a^ |  |
| Any mental illness | 1.27 (1.24-1.30) | 1.23 (1.19-1.26) |  |
| Depression | 1.29 (1.23-1.34) | 1.22 (1.17-1.28) |  |
| Bipolar disorder | 1.12 (1.05-1.19) | 1.11 (1.04-1.19) |  |
| Psychotic disorder | 1.29 (1.19-1.40) | 1.28 (1.17-1.39) |  |
| Stress and anxiety disorder | 1.28 (1.23-1.32) | 1.24 (1.20-1.28) |  |
| Personality disorder | 1.32 (1.26-1.39) | 1.30 (1.23-1.37) |  |
| Intentional self-harm | 1.24 (1.16-1.33) | 1.22 (1.13-1.30) |  |

^a^Hazard ratio for preterm vs. term birth, adjusted for age, comorbidity, substance use disorder, gestational diabetes, severe maternal morbidity, cesarean section, multiple birth, fetal congenital anomaly, socioeconomic deprivation, rurality, and time period.

**Table S4** Association between severity of preterm birth at first delivery and maternal mental illness hospitalization

|  | Hazard ratio  (95% confidence interval) | | |
| --- | --- | --- | --- |
|  | Unadjusted | Adjusted^a^ | |
| Preterm birth, weeks |  | |  |
| <37 | 1.27 (1.24-1.30) | | 1.23 (1.19-1.26) |
| <28 | 1.28 (1.16-1.42) | | 1.24 (1.13-1.37) |
| 28-31 | 1.32 (1.22-1.44) | | 1.22 (1.13-1.33) |
| 32-36 | 1.26 (1.23-1.30) | | 1.23 (1.19-1.26) |
| ≥37 weeks | Reference | | Reference |

^a^ Hazard ratio for preterm vs. term birth, adjusted for age, comorbidity, substance use disorder, gestational diabetes, severe maternal morbidity, cesarean section, multiple birth, fetal congenital anomaly, socioeconomic deprivation, rurality, and time period.
